# Depletion of neural stem cells from the subventricular zone of adult mouse brain using cytosine b‐Arabinofuranoside

**DOI:** 10.1002/brb3.404

**Published:** 2015-10-15

**Authors:** Amir Ghanbari, Tahereh Esmaeilpour, Soghra Bahmanpour, Mohammad Ghasem Golmohammadi, Sharareh Sharififar, Hassan Azari

**Affiliations:** ^1^ Neural Stem Cell and Regenerative Neuroscience LaboratoryDepartment of Anatomical SciencesShiraz School of MedicineShiraz University of Medical SciencesShirazIran; ^2^ Department of Anatomical SciencesArdabil University of Medical SciencesArdabilIran; ^3^ Department of Physical TherapyCollege of Public Health and Health ProfessionsUniversity of FloridaGainesvilleFlorida; ^4^ Neural Stem Cell and Regenerative Neuroscience LaboratoryShiraz Stem Cell InstituteShiraz University of Medical SciencesShirazIran

**Keywords:** Ara‐C infusion, neural stem cell depletion, neural colony‐forming cell assay, neurosphere assay, subventricular zone

## Abstract

**Introduction:**

Neural stem cells (NSCs) reside along the ventricular axis of the mammalian brain. They divide infrequently to maintain themselves and the down‐stream progenitors. Due to the quiescent property of NSCs, attempts to deplete these cells using antimitotic agents such as cytosine b‐Aarabinofuranoside (Ara‐C) have not been successful. We hypothesized that implementing infusion gaps in Ara‐C kill paradigms would recruit the quiescent NSCs and subsequently eliminate them from their niches in the subventricular zone (SVZ).

**Methods:**

We infused the right lateral ventricle of adult mice brain with 2% Ara‐C using four different paradigms—1: one week; 2: two weeks; 3, 4: two weeks with an infusion gap of 6 and 12 h on day 7. Neurosphere assay (NSA), neural colony‐forming cell assay (N‐CFCA) and immunofluorescent staining were used to assess depletion of NSCs from the SVZ.

**Results:**

Neurosphere formation dramatically decreased in all paradigms immediately after Ara‐C infusion. Reduction in neurosphere formation was more pronounced in the 3rd and 4th paradigms. Interestingly 1 week after Ara‐C infusion, neurosphere formation recovered toward control values implying the presence of NSCs in the harvested SVZ tissue. Unexpectedly, N‐CFCA in the 3rd paradigm, as one of the most effective paradigms, did not result in formation of NSC‐derived colonies (colonies >2 mm) even from SVZs harvested 1 week after completion of Ara‐C infusion. However, formation of big colonies with serial passaging capability, again confirmed the presence of NSCs.

**Conclusions:**

Overall, these data suggest Ara‐C kill paradigms with infusion gaps deplete NSCs in the SVZ more efficiently but the niches would repopulate even after the most vigorous kill paradigm used in this study.

## Introduction

Neural stem cells (NSCs) are residing in niches along the ventricular neuraxis of the mammalian nervous system (Craig et al. 1996; Golmohammadi et al. 2008; Mirzadeh et al. 2008; Shen et al. 2008). They are capable of self‐renewal, prolonged cell division, and generating a large number of progeny (Reynolds and Weiss 1992). Previous studies have demonstrated three main cell types in the adult subventricular zone (SVZ) stem cell niche; namely, type B NSCs (glial fibrillary acidic protein (GFAP^+^) expressing cells) that give rise to type C transit amplifying cells (GFAP^−^/Dlx2^+^), which in turn generate type A neuroblast (GFAP^−^/Dlx2^+^/doublecortin (DCX)^+^) cells (Doetsch et al. 1999b; Riquelme et al. 2008; Chojnacki et al. 2009) migrating through a channel of interwoven astrocytes, the rostral migratory stream (RMS), to the olfactory bulb. The SVZ niche is separated from the cerebrospinal fluid (CSF) of the ventricles via a thin layer of multiciliated ependymal cells. Ependymal cells not only act as a physical barrier and a sensor of CSF components through coupling with SVZ astrocytes but also secrete proneurogenic factors such as Noggin to create a favorable neurogenic environment (Lim et al. 2000). Some of the type B cells have long processes intercalating between adjacent ependymal cells to assess the ventricular area (Doetsch et al. 1999a; Silva‐Vargas et al. 2013; Codega et al. 2014). In contact with the ventricle, these processes express a primary cilium that might function for transduction of signals in the CSF. Away from the ventricular side, the niche is related to a dense network of vessels with laminin‐rich basal lamina (Mercier et al. 2002; Silva‐Vargas et al. 2013). Cellular states of quiescence, proliferation, differentiation in SVZ niche is finely tuned via multiple mechanisms including the inherent genetic state of the niche cells and the signals arriving from the microenvironment including the CSF, niche blood vessels, surrounding neural networks via axonal terminals and interaction of niche resident cells (Doetsch et al. 1997; Silva‐Vargas et al. 2013). Interestingly, among the cell content of the stem cell niche in the SVZ, the NSCs (type B) are quiescent and divide infrequently to maintain the pool of stem cells and the down‐stream progenitors through symmetric or asymmetric divisions (Morshead et al. 1994; Riquelme et al. 2008). This characteristic reduces the possibility of mutations in the genome of long‐lived stem cells (Reya et al. 2001). Experiments on in vivo activation and/or depletion of the NSCs and their progeny have largely increased our understanding of niche microenvironment, cellular diversity, and behavior. Antimitotic drug cytosine b‐Aarabinofuranoside (Ara‐C) can actively eliminate dividing cells. Researchers used Ara‐C treatment to eliminate neural stem and progenitor cells from the SVZ stem cell niches but these attempt were not successful to eliminate the entire pool of NSCs mainly due to their quiescent property at the time of antimitotic drug infusion and also because of applying short term (3–7 days) continuous Ara‐C infusion paradigms (Morshead et al. 1994; Pastrana et al. 2009; Codega et al. 2014; Sachewsky et al. 2014). In a study by Fiona Doetsch et al. after AraC infusion for 6 days and examining the whole‐mount preparation of the SVZ at 0 and 12 h post infusion, they could only see activated NSCs expressing minichromosome maintenance 2 (MCM2), a marker indicating the initiation of DNA replication (G_1_ to S phase), at 12 h post infusion (Doetsch et al. 1999a). In a separate study, after infusing the mice with AraC for 6 days, they injected BrdU 1 h before dissection and evaluated reappearance of proliferative cells in a course of 0–14 days after AraC removal and showed that BrdU‐positive cells appeared 6–12 h after pump removal and were scattered throughout the SVZ (Doetsch et al. 1999b). Similarly, Pastrana e al showed that starting from a few hours after removal of the antimitotic drug, the quiescent NSCs activate for a short window of time to repopulate depleted progeny in the niche (Pastrana et al. 2009). In this study, we aimed to eliminate NSCs by their activation via implementing Ara‐C infusion gaps. Accordingly, besides a short term (7 days) and a long term (14 days) continuous Ara‐C infusion, we also employed two other kill paradigms for a total 14 days infusion with a 6 and 12 h drug infusion gaps on day 7. The success of the kill paradigm were assessed by the neurosphere assay (NSA) to compare the number of the remaining actively dividing cells in different kill paradigms and the neural colony‐forming cell assay (N‐CFCA) to ensure the presence or absence of NSCs in the best kill paradigm.

## Materials and Methods

### Animals and experimental groups

Five‐week‐old male C57‐BL6 mice were housed five per cage and kept under a normal light–dark cycle (12 h light and 12 h dark) in an air‐conditioned room with a temperature of 23 ± 1°C in the animal facility of Shiraz University of Medical Sciences, with free access to food and water. The Ethics Committee for Animal Experiments at Shiraz University of Medical Sciences approved all animal procedures that were performed based on the international guidelines for biomedical research using animals. At the beginning of the study, animals (*N* = 42) were randomly divided into control, sham, and experimental groups. In the control group, the animals were killed without any intervention, their brains were removed and the right and left SVZ were separately microdissected and cultured. In the sham group, the animals received right intraventricular infusion of 0.09% saline for 7 days before culturing their SVZ. In the experimental groups, the animals received right intraventricular infusion of 2% Ara‐C for 7, 14, and 14 days with an infusion gap of 6 and 12 h on day 7 (7‐6 h‐7 and 7‐12 h‐7 paradigms). Animals from the experimental groups were killed immediately or 1 week after the completion of Ara‐C treatment. Animals from each group were first anesthetized with isoflurane and then underwent a cervical dislocation to harvest the brain tissue for culture or perfused with cold 4% paraformaldehyde to preserve the tissue for immunofluorescent analysis.

### Osmotic mini‐pump surgical implantation

Animals in sham and experimental groups were anesthetized by 4% isoflurane and subsequently secured in a stereotaxic apparatus (Stoelting, Kiel, WI). While the animal head was positioned horizontally and the anesthesia was maintained by 1.5–2% isoflurane via a facemask, eye ointment was applied and scalp was shaved and cleaned with alcohol and betadine. Then, a midline incision was applied and the skin was retracted to expose the skull. Using a sterile dental drill a 1 mm burr hole was made in right side of the skull and the cannula of a brain kit 3 (Brain Infusion Kit, Durect, Cupertino, CA) was inserted into the right lateral ventricle and secured via Loctite 454 Cyanoacrylate Adhesive (Durect, Cupertino, CA) at the following coordinate according to Paxinos mouse brain Atlas: Anterior‐Posterior (AP): −0. 2, Medial‐Lateral (ML): +0. 9 from the bregma point and Dorsal‐Ventral: +2.7 from the top of skull. The cannula was attached via a connecting tube to an osmotic mini‐pump (model 1007D; flow rate 0.5 L/h, 7 d; Durect) containing 100 μL of sterile saline or 2% Ara‐C solution. The pump reservoir was implanted under the skin on the back of the animal and was hydrated by subcutaneous injection of 1 mL sterile saline to initiate pumping. Animals were given Buprenorphine at 0.5 mg/kg every 12 h for 48 h to reduce postoperative pain. In 14 days treatment groups the empty pumps on day 7 were replaced with newly filled pumping reservoirs immediately after removal of the empty pump to ensure continuous Ara‐C infusion or 6 and 12 h later to cause a 6 and 12 h gap in Ara‐C infusion in order to activate the dormant NSCs.

### Neurosphere assay, sphere‐forming frequency and differentiation of neural stem cells

A thin layer of tissue containing the SVZ of the lateral ventricles, extending from the olfactory bulb to the crossing of the anterior commissure, was carefully microdissected from each brain and plated in neurosphere culture as described elsewhere (Azari et al. 2010; Siebzehnrubl et al. 2011). Briefly, tissue from the left and right SVZ was removed separately and cut into very small pieces and digested with 0.05% Trypsin‐EDTA (Gibco, Carlsbad, CA) for 5–7 min at 37°C. After enzymatic digestion, soybean trypsin inhibitor (Sigma, St. Louis, MO) was added and the cell suspension was mixed gently and centrifuged at 110 g. The cell pellet was resuspended in neurocult‐neural stem cell (STEMCELL Technologies, Vancouver, Canada) medium (500 μL) and mechanically dissociated to achieve a single‐cell suspension. The cell suspension was spun (110 g, 5 min) to remove the supernatant. Finally, the cells harvested from each SVZ were resuspended in complete neurosphere medium (5 mL) supplemented with EGF (20 ng/mL; STEMCELL Technologies), bFGF (10 ng/mL; STEMCELL Technologies) and heparin (2 μg/mL; Sigma, St. Louis, MO) and incubated in a T25 flask (NUNC) in a humidified incubator with 5% CO_2_ for 8 days. The number of neurospheres were counted and compared between different treatment groups. To evaluate the capability of the resulting neurospheres for long‐term passage, the neurospheres were first dissociated into single cells and replated in complete neurosphere medium supplemented with growth factors for 8 days. Every 8 days the spheres were harvested and replated in fresh medium and the total number of harvested cells was recorded per each passage. To evaluate the differentiation capabilities of the neurospheres, single cell harvested from dissociated neurospheres were plated in neuroblast assay differentiation culture (Azari et al. 2012) for 8 days and immunostained for neuronal marker ß‐III tubulin and glial marker glial fibrillary acidic protein (GFAP).

### Neural colony‐forming cell assay and stem cell frequency

The harvested SVZ tissue from each brain of the selected groups (control animals and animals treated with 7‐6 h‐7 Ara‐C kill paradigm) was processed in the same way as described for neurosphere assay and finally cultured in a semisolid collagen‐based medium known as the neural colony‐forming cell assay (N‐CFCA, STEMCELL Technologies, Vancouver, Canada) for 28 days to discriminate stem cells (colonies equal or bigger than 2 mm in diameter) from the progenitor cells (colonies less than 2 mm) based on their proliferative potential (Louis et al. 2008; Azari et al. 2011). Culture plates were fed every week with medium supplemented with EGF and b‐FGF as per manufacturer's instruction. After 28 days, colonies were counted and their size was measured.

### Immunofluorescence staining

Control animals and animals treated with 7‐6 h‐7 Ara‐C kill paradigm were first anesthetized by 4% isoflurane and then perfused transcardially with 0.09% saline and 4% cold paraformaldehyde in PBS (pH= 7.4). Brains were postfixed by immersion in the same fixative overnight and then immersed in 30% sucrose solution for 2 days. Serial 10 μm cryosections were prepared form the SVZ of lateral ventricles for immunofluorescence staining. Immunofluorescence staining was performed using the standard protocol by overnight incubation of samples in right combination of rabbit anti‐GFAP (DakoCytomation), guinea pig anti‐DCX (Millipore), rabbit anti‐ki67 (Millipore) and mouse anti‐Nestin (Millipore, MAB353) primary antibodies in 0.03% PBS‐Triton‐×100 supplemented with 5% normal goat serum. After three washing with PBS, the primary antibodies were detected by incubation of samples for 1 h at room temperature in 0.03% PBS‐Triton‐×100 supplemented with 5% normal goat serum containing secondary antibodies conjugated with Alexa flour 488 and 568 and DAPI for nuclear staining. Seven representative sections with an approximately 350 micron interval from the entire SVZ extending from anterior commissure to the beginning of the olfactory bulb were carefully monitored for each staining and the number of immunostained cells was quantified in each section. Then, an average number/section was calculated for each animal. Data from three animals/group were presented here as mean ± SEM. Representative images for each staining were also captured using a fluorescent microscope (Nikon Eclipse E600, Fall River, MA) and the images were merged using Adobe Photoshop CS4 software.

### Statistics analysis

Data analysis was performed using one‐way ANOVA or Student's *t*‐tests as appropriate (Prism 5; Graphpad Software Inc., San Diego, CA). Results were expressed as mean ± SEM and the level of significance was set at a *P*‐value of less than 0.05.

## Results

### Intraventricular Ara‐C infusion depletes dividing cells in the subventricular zone

**Figure 1:**
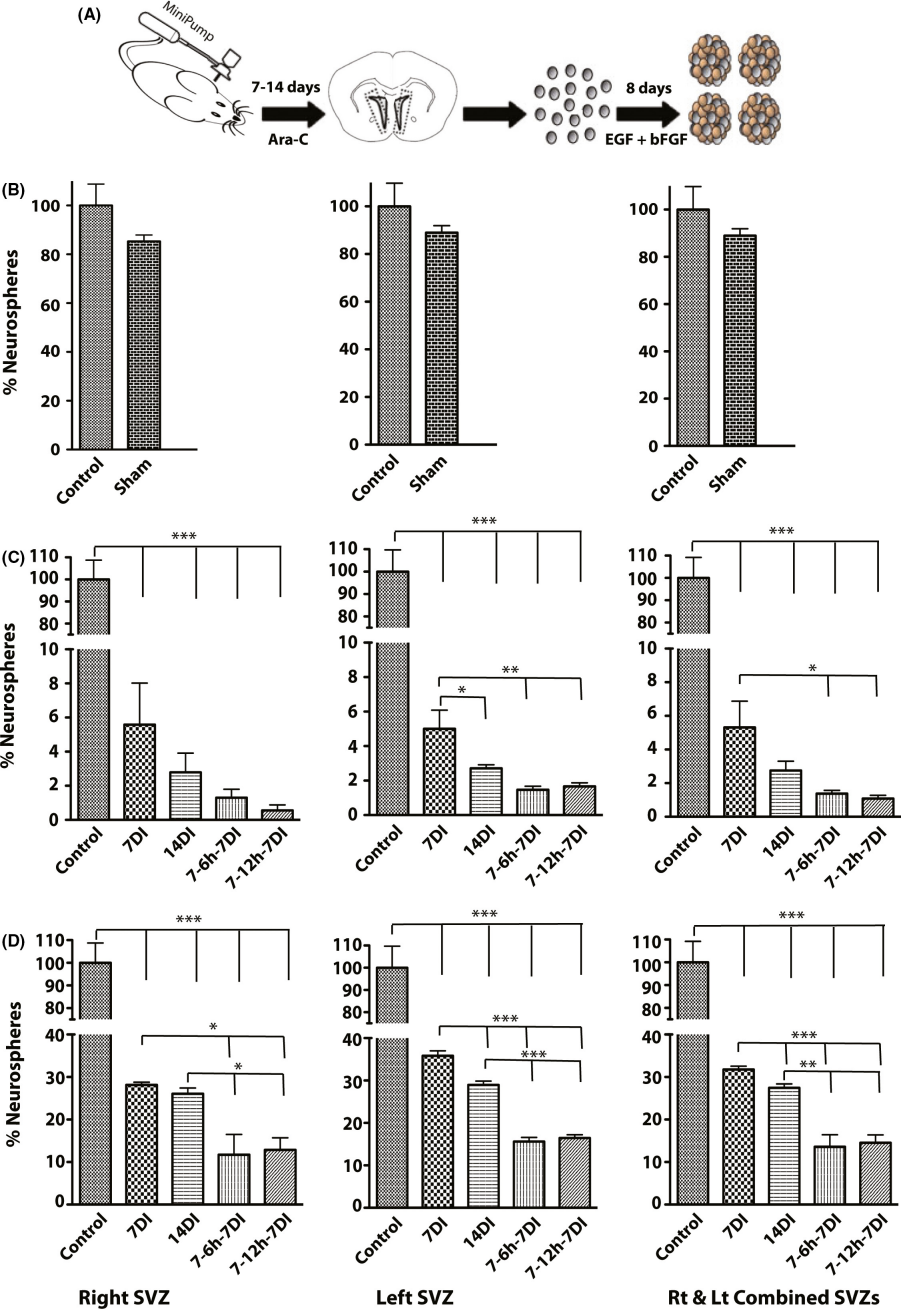
Study design, neurosphere assay and comparing the number of neurospheres in different Ara‐C kill paradigms (A–D). A: Schematic diagram showing the study design and the neurosphere assay. B: Relative number of neurospheres per right, left and combined subventricular zone (SVZ) tissue in control and sham (saline infused) groups. No statistically significant difference was observed. C: Relative number of neurospheres per right, left and combined SVZ tissue in control versus different Ara‐C kill paradigms immediately after completion of Ara‐C infusion. As evident, neurosphere formations reduced significantly in all Ara‐C kill paradigms comparing to the control group (***P < 0.0001). This reduction was significantly higher in 14 days, 7‐6 h‐7, and 7‐12 h‐7 infusion paradigms (**P < 0.001, *P < 0.05) comparing to 7 days infusion paradigm. D: Relative number of neurospheres per right, left, and combined SVZ tissue in control versus different Ara‐C kill paradigms 1 week after completion of Ara‐C infusion. Interestingly, neurosphere formation frequency increased to 10–30% of control value but still is significantly lower in all Ara‐C groups comparing to the control group (***P < 0.0001). 14 days, 7‐6 h‐7, and 7‐12 h‐7 infusion paradigms resulted in a more pronounced reduction (***P < 0.0001, **P < 0.001, *P < 0.05) as compared to the 7 days infusion paradigm. All values are mean ± SEM relative to control. *N* = 3 independent experiments.

To evaluate the efficiency of different kill paradigms in depleting dividing cells in the subventricular zone (SVZ) of the lateral wall of the lateral ventricles in adult mouse brain, we employed the neurosphere assay (NSA), which is routinely used as a valid in vitro readout of the overall neural stem and progenitor cells number in a given part of the nervous tissue (Azari et al. 2010; Siebzehnrubl et al. 2011) (Fig. 1A). All values are presented as a percentage of their corresponding control values. Moreover, the mean absolute neurosphere number per each condition is shown in Table 1. Comparing control‐ and sham‐treated groups revealed that 1 week of continuous saline infusion did not significantly decrease the mean neurosphere‐forming frequency (NFF) in harvested cells from the right, left, and combined SVZs, respectively (85.29 ± 2.6% vs. 100 ± 8.8% in Rt SVZ, 88.96 ± 2.91% vs. 100 ± 9.7% in Lt SVZ and 92 ± 5.64% vs. 100 ± 9.7% in combined SVZs in saline vs. control group, respectively, Fig. 1B).

**Table 1:** Absolute neurosphere number/condition after plating cells in culture for 8 days

Groups	Control	Sham	7 Days	14 Days	7‐6 h‐7 Days	7‐12 h‐7 Days	7 Days Post 7 Days	7 Days Post 14 Days	7 Days Post 7‐6 h‐7 Days	7 Days Post 7‐12 h‐7 Days
Right (SVZ)	179 ± 15.72	161.3 ± 7.51	10 ± 4.36	5 ± 2	2.33 ± 0.88	1 ± 0.58	50.33 ± 1.20	46.67 ± 2.40	21 ± 8.54	23 ± 5.13
Left (SVZ)	160 ± 15.53	150.7 ± 12.78	8 ± 1.73	4.33 ± 0.33	2.33 ± 0.33	2.66 ± 0.33	57.33 ± 1.85	46.33 ± 1.45	25 ± 1.52	26.33 ± 1.2
Combined (SVZs)	339 ± 31.18	312 ± 19.14	18 ± 5.29	9.33 ± 1.85	4.66 ± 0.66	3.66 ± 0.66	107.7 ± 2.72	93 ± 3.21	46 ± 9.7	49.33 ± 6.17

In experimental groups, the mean NFF of the harvested cells from right, left, and combined SVZs significantly decreased in all kill paradigms immediately after completion of Ara‐C treatment comparing to the control (5.6 ± 2.4%, 2.8 ± 1.1%, 1.3 ± 0.5%, 0.56 ± 0.32% in Rt SVZ; 5 ± 1.1%, 2.7 ± 0.2%, 1.46 ± 0.2%, 1.67 ± 0.32% in Lt SVZ; 5.3 ± 1.56%, 2.75 ± 0.55%, 1.37 ± 0.19%, 1.1 ± 0.2% in combined SVZs in 7, 14, 7‐6 h7, 7‐12 h‐7 days infusion paradigms, respectively). All these values were significantly different to the control (Fig. 1C, *P* < 0.0001). Moreover, comparing different Ara‐C treatment paradigms revealed that neurosphere reduction was significantly higher in Lt SVZ of 14 days, 7‐6 h‐7, and 7‐12 h‐7 paradigms and in combined SVZs of 7‐6 h‐7 and 7‐12 h‐7 paradigms comparing to 7 days paradigm (Fig. 1C, *P* < 0.05).

**Figure 2:**
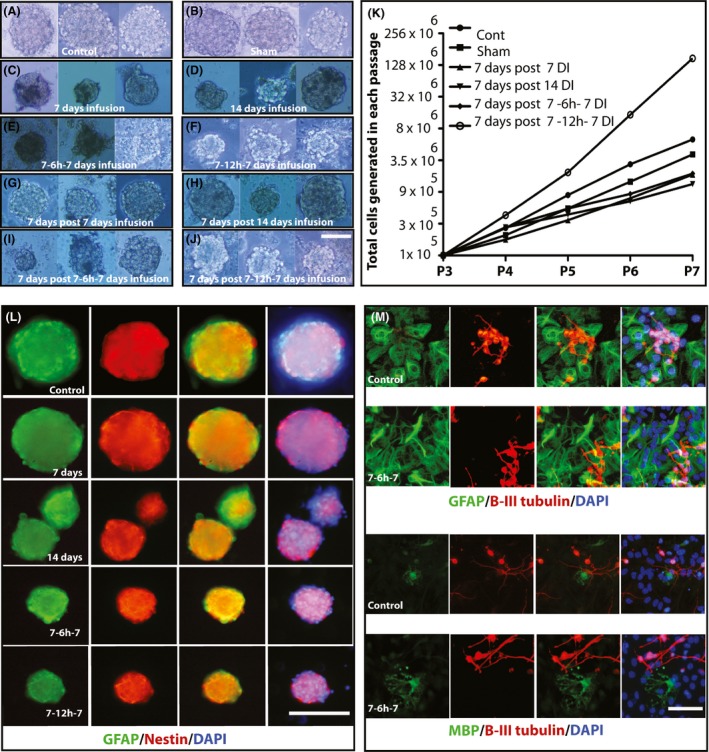
Representative neurosphere pictures from different treatment groups, serial passaging and immunostaining of neurospheres and differentiated neural stem cells (A–M). A, B: Neurospheres grown after 8 days in culture from the SVZ tissue harvested from control and sham groups, respectively. No differences could be noticed in their relative size and morphology. C‐F: Neurospheres grown after 8 days in culture from the SVZ of animals harvested immediately after completion of Ara‐C treatments from different Ara‐C kill paradigms. As evident, neurospheres are smaller in size and appear more compact comparing to the control group. G–J: Neurospheres grown after 8 days in culture from the SVZ of animals harvested 1 week after completion of Ara‐C treatments from different Ara‐C kill paradigms. As shown, neurospheres are more similar to the control group but still are smaller in size. K: Neurospheres harvested from control, sham, and all Ara‐C treatment groups that were harvested 1 week after completion of Ara‐C treatment could be serially passaged in culture (*N* = 3 independent experiments for each condition). L: Representative neurospheres from the control and different Ara‐C kill paradigms 7 days after completion of Ara‐C infusion. Neurospheres were immunostained for GFAP, Nestin and counterstained with DAPI. M: Representative immunostaining for neuronal (ß‐III tubulin), astrocytic (glial fibrillary acidic protein, GFAP) and oligodendroglial (myelin basic protein, MBP) differentiation of the harvested neural stem cells from the control and 7‐6 h‐7 Ara‐C kill paradigm 7 days after completion of Ara‐C infusion. Scale bars represent 100 μm in neurosphere pictures and 50 μm in differentiated NSCs pictures.

Morphological assessment showed that neurospheres resulted from the control and sham groups were big, round, and not compact (Fig. 2A, B). In contrast, neurospheres in Ara‐C‐treated groups appeared small, compact and sometimes irregular‐shaped (Fig. 2C–F). To evaluate whether these neurospheres were derived from NSCs or progenitor cells, we collected all the resulting neurospheres from the control, sham and Ara‐C kill paradigms, dissociated them into single cells and replated in neurosphere culture to assess their ability for long‐term passaging. Interestingly, except for the control and sham group, the cells from all Ara‐C groups failed to expand beyond 1–2 passages in vitro.

Resulting neurospheres from the control group expressed glial fibrillay acidic protein (GFAP) and nestin as neural stem and progenitor cell markers and generated neuronal and glial cells upon differentiation as evidenced by expression of ß‐III tubulin, GFAP, and myelin basic protein (MBP) (Fig. 2L, M).

### The NSC niche of the SVZ repopulates 1 week after completion of intraventricular Ara‐C infusion

To test which Ara‐C kill paradigm has a longer lasting effects in terms of neural stem and progenitor cell reduction, we prepared a second set of animals with the same Ara‐C kill paradigms as described before and let them survive for a week after completion of Ara‐C infusion before culturing them in neurosphere assay (NSA). Although the mean NFF of all Ara‐C treatment groups increased (varying from 10 to 30% of the matched control groups), these values were still significantly lower comparing to their corresponding control groups (28.12 ± 0.67%, 26.1 ± 1.34%, 11.73 ± 4.8%, 12.85 ± 2.88% in Rt SVZ; 35.83 ± 1.16%%, 28.96 ± 0.91%, 15.63 ± 0.95%, 16.46 ± 0.75% in Lt SVZ; 31.76 ± 0.8%, 27.43 ± 0.95%, 13.57 ± 2.86%, 14.55 ± 1.82% in combined SVZs in 7, 14, 7‐6 h‐7, 7‐12 h‐7 days infusion paradigms, respectively, Fig. 1D, *P* < 0.0001). Mean absolute neurosphere number per each condition is shown in Table 1.

Moreover, statistical analysis between different Ara‐C treatment groups at 1 week after completion of Ara‐C infusion revealed that mean NFF was significantly lower in Rt SVZ of 7‐6 h‐7 and 7‐12 h‐7 paradigms comparing to 7 and 14 days paradigms (Fig. 1D, *P* < 0.05) and in Lt SVZ of 14 days, 7‐6 h‐7, and 7‐12 h‐7 paradigms comparing to 7 days paradigm and also 7‐6 h‐7 and 7‐12 h‐7 paradigms comparing to 14 days paradigm (Fig. 1D, *P* < 0.0001). Similarly, these values were also significantly lower in combined SVZs of 7‐6 h‐7 and 7‐12 h‐7 paradigms comparing to 14 and 7 days paradigm (Fig. 1D, *P* < 0.0001, *P* < 0.05, respectively).

Morphologically, neurospheres resulted from Ara‐C‐treated groups 1 week after completion of Ara‐C infusion (Fig. 2G–J) were more similar to the control and sham (Fig. 2A, B) in terms of their size, shape and compactness. Serial passaging of these neurospheres showed that all Ara‐C groups could successfully expand in vitro at a comparable level to the neurospheres from control and sham groups (Fig. 2K). Resulting neurospheres from the Ara‐C groups expressed glial fibrillay acidic protein (GFAP) and nestin as neural stem and progenitor cell markers and generated neuronal and glial cells upon differentiation as evidenced by expression of ß‐III tubulin, GFAP, and MBP (Fig. 2L, M).

### Neural stem cells (NSCs) escape Ara‐C kill or re‐emerge from a developmentally more primitive cell source even in the most rigorous Ara‐C kill paradigm

**Figure 3:**
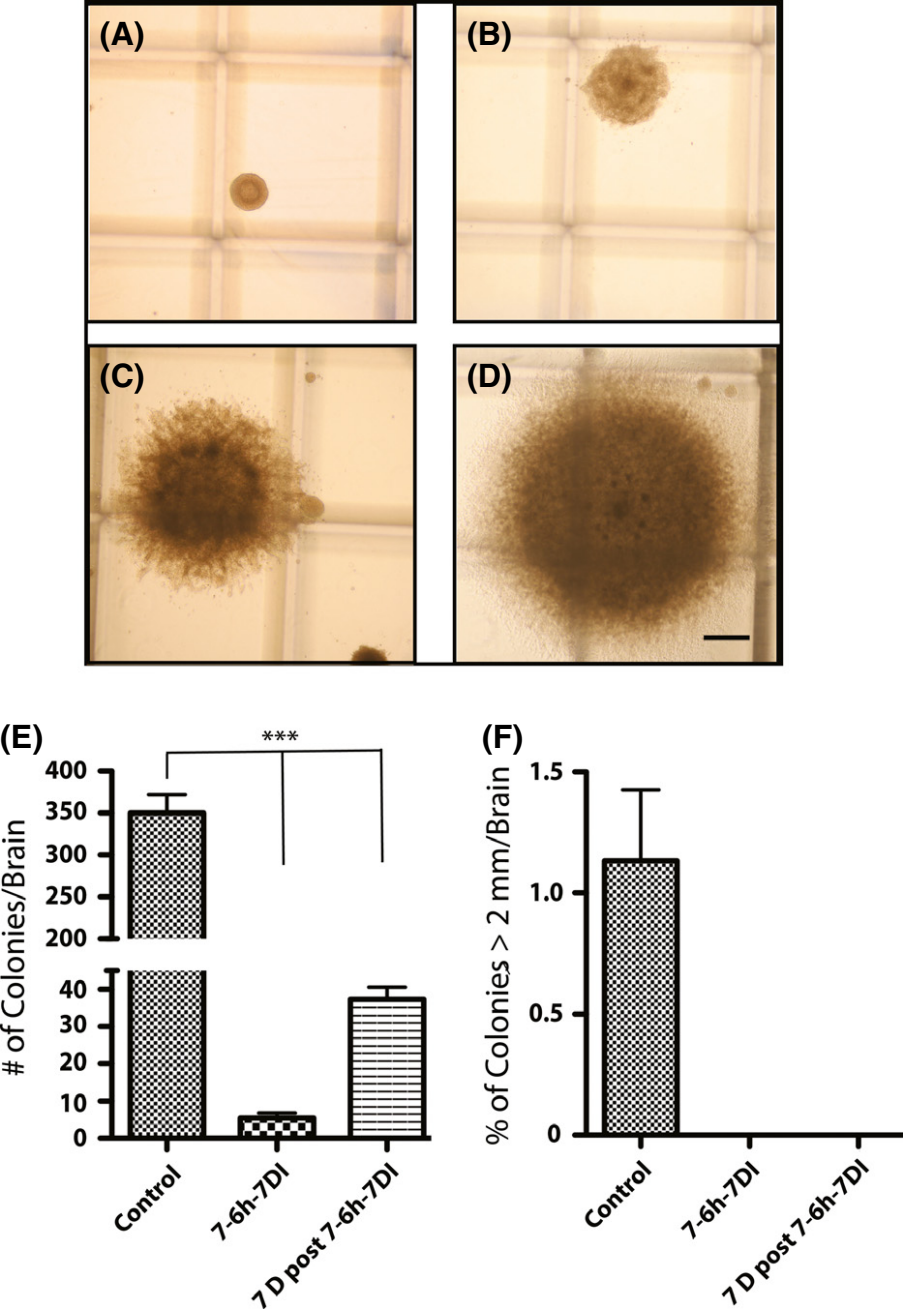
Representative colonies grown in N‐CFCA and the colony‐forming frequency in control and 7‐6 h‐7 Ara‐C kill paradigm animals immediately and 1 week after completion of Ara‐C infusion (A–F). A–D: Representative colonies are shown at different size categories; A: less than 0.5 mm in diameter, B: 0.5–1 mm, C: 1–2 mm, and D: equal or bigger than 2 mm in diameter. A, B, and C are progenitor‐derived colonies and D is a NSC‐derived colony. E: Colony‐forming frequency per brain (combined Rt & Lt SVZs) in control and 7‐6 h‐7 Ara‐C kill paradigm immediately and 7 days after completion of Ara‐C treatment. The overall number of colony was significantly higher in the control group (***P < 0.0001). F: Frequency of NSC‐derived colonies (equal or bigger than 2 mm in diameter) as a percentage of total colonies in control and 7‐6 h‐7 Ara‐C kill paradigm immediately and 7 days after completion of Ara‐C treatment. No colonies of equal or bigger than 2 mm were grown from SVZ N‐CFCA culture in 7‐6 h‐7 Ara‐C kill paradigm. Scale bars represent 500 μm in colony pictures. All values are mean ± SEM. *N* = 3 independent experiments.

One week after completion of Ara‐C infusion the mean NFF increased in all kill paradigms and reached to 10–30% of the control value. This implies reactivation of NSCs in niches after AraC removal and generation of new proliferating progeny that resulted in more neurosphere formation upon culture. To confirm the presence of NSCs in the harvested SVZs of the adult mouse brain after Ara‐C treatment, we employed the neural colony‐forming cell assay (N‐CFCA) that can detect these cells based on their long‐term proliferation capabilities (Louis et al. 2008; Azari et al. 2011). We harvested SVZ tissue from the animals treated with 7‐6 h‐7 Ara‐C kill paradigm and compared its stem cell frequency with an untreated control group. 7‐6 h‐7 Ara‐C kill paradigm was chosen as it appeared as one of the most efficacious paradigm in terms of reducing neurosphere formation in animals killed immediately and 1 week after Ara‐C treatment completion with no statistically significant difference to 7‐12 h‐7 Ara‐C kill paradigm. After 28 days of culturing the harvested SVZs cells in a semisolid collagen‐based medium, the total number of colonies (representing the number of stem and progenitor cells collectively) and also colonies equal or bigger than 2 mm in diameter (representing the number of NSCs) were quantified for each group (Fig. 3A–D). While the total number of colonies was 350 ± 21.67 per combined SVZs in control group, this measure was 5.5 ± 1.26 (1.57 ± 0.36% of the control) and 37.33 ± 3.28 (10.66 ± 0.94% of the control) in 7‐6 h‐7 Ara‐C kill paradigm immediately and 1 week after completion of Ara‐C treatment, respectively (Fig. 3 E). Statistical analysis showed that the number of colonies was significantly lower in Ara‐C‐treated groups compared to the control group (*P* < 0.0001).

While the number of NSC‐derived colonies per combined SVZs was 4 ± 1.15 (1.14 ± 0.32% of the total colonies) in control group, we could not found any NSC‐derived colonies (colonies equal or bigger than 2 mm in diameter) in 7‐6 h‐7 Ara‐C kill paradigm immediately and 1 week after completion of Ara‐C treatment, respectively (Fig. 3F). Furthermore, while in 7‐6h‐7 Ara‐C kill paradigm immediately after completion of Ara‐C treatment the majority of colonies were less than 1 mm in diameter, we could find a few colonies between 1–1.5 mm (28.6 ± 2.4% of total colonies) and one colony between 1.5–2 mm (0.9 ± 0.9% of total colonies) 1 week after completion of Ara‐C treatment. Although we could not find NSC‐derived colonies, choosing 19 colonies close to 1.5 mm in diameter showed that four of them (including the colony with the diameter between 1.5–2 mm) could be serially passaged implying the presence of NSCs in those colonies (data not shown).

### Immunofluorescent analysis of the Ara‐C‐treated SVZ immediately after completion of 7‐6 h‐7 Ara‐C kill paradigm

**Figure 4:**
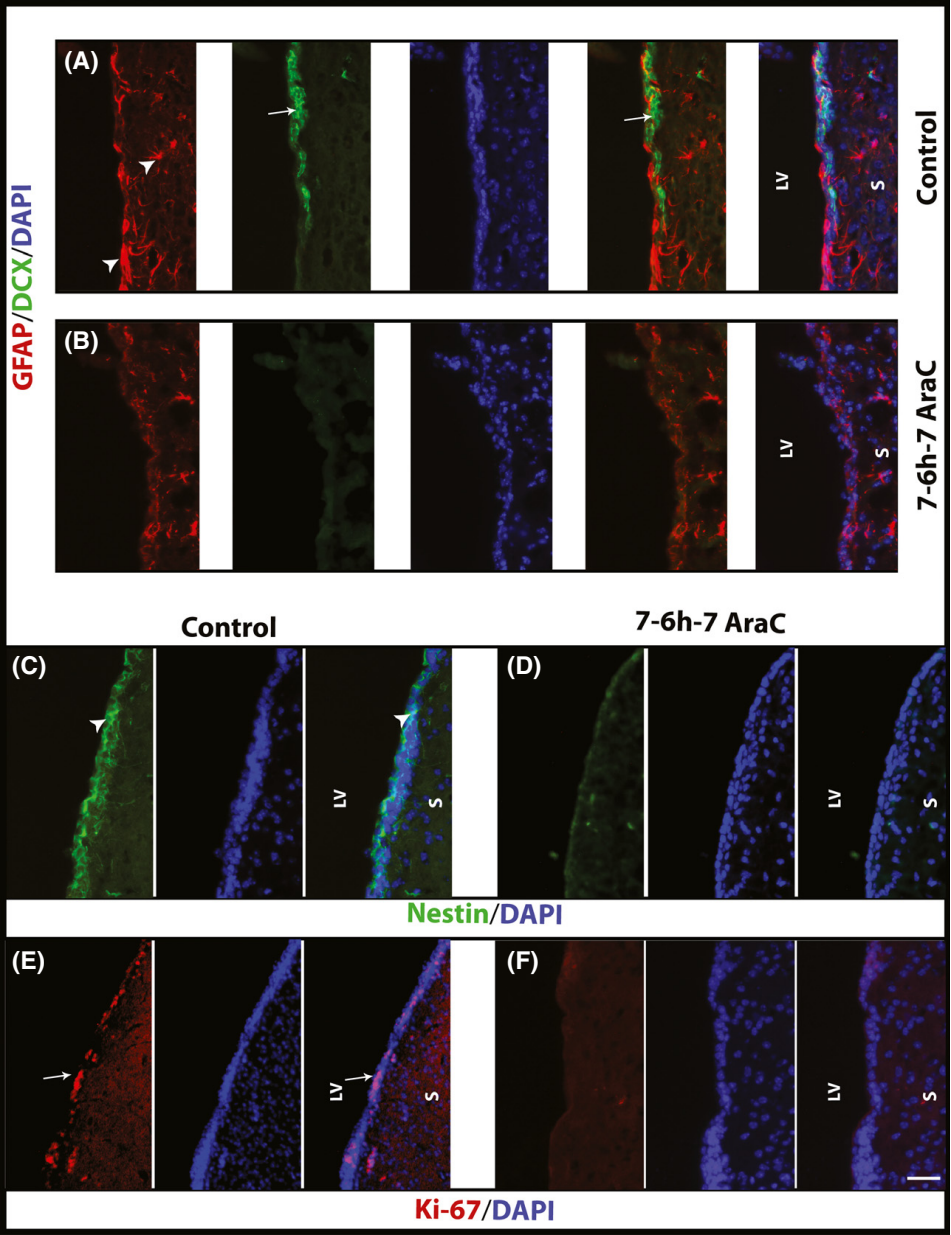
Representative immunofluorescent images from the subventricular zone (SVZ) of control and Ara‐C treated (7‐6 h‐7 Ara‐C kill paradigm) animals immediately after completion of Ara‐C infusion (A–E). All sections are counterstained with DAPI (blue) to visualize the nuclei. A, B: GFAP (red) and doublecortin (DCX, green) stained sections from the SVZ of control (A) and Ara‐C (B) groups. GFAP staining could be observed in both groups. These cells and their processes (*arrow heads*) could sharply be detected in both ad‐luminal (immediately under the lining of the ventricle) and ab‐luminal (close to striatal parenchyma) parts of the SVZ in control group. DCX staining was limited to the control group and could be visualized as clusters of cells (*arrows*) surrounded by GFAP‐stained cell processes. C, D: Nestin immunostaining of representative sections from the SVZ of control (C) and Ara‐C (D) groups. As evident, nestin staining could be observed as layers of intense green‐stained cells (*arrow heads*) lining the lateral wall of the ventricle in control group. This staining was mainly lost in the Ara‐C treatment group and only a faint trace of nestin immunoreactivity could be detected in the lining of the lateral ventricle. E, F: Ki‐67 immunostaining of control (E) and Ara‐C (F) treated SVZs. While many Ki‐67 immunoreactive proliferative cells (*arrows*) were detected either as clusters or individual cells in control SVZ, these cells were almost depleted from the Ara‐C treated SVZs. LV: Lateral Ventricle, S: Striatum. Scale bar represents 50 μm.

To evaluate the effectiveness of 7‐6 h‐7 Ara‐C kill paradigm in depletion of stem and progenitor cells and assess the cellular composition of the SVZ immediately after completion of Ara‐C treatment, we immunostained representative sections from the control as well as the Ara‐C‐treated animals with different markers such as GFAP and nestin (NSC markers), doublecortin (DCX, a marker of neuroblast cells), and Ki‐67 (a marker of actively dividing cells). Close assessment of the SVZ region in the lateral wall of the lateral ventricle extending from the level of anterior commissure to the olfactory bulb under a fluorescent microscope showed that GFAP staining was present in both the control and Ara‐C‐treated animals. Interestingly, while this staining was very intense in control group and the cells could be observed in both the SVZ and surrounding regions with detectable cell bodies and processes, in Ara‐C‐treated group the GFAP staining was mainly found in the neighboring striatum while extending some processes toward the SVZ (Fig. 4A, B). We could count an average of 6.43 ± 0.93 GFAP‐positive cells/section in the SVZ of the animals killed immediately after 7‐6 h‐7 Ara‐C kill paradigm, whereas this number was 29.43 ± 4.53 in control animals. Furthermore, DCX positive neuroblast cells were only found in the control animals in patches surrounded by the GFAP‐positive cells (24.95 ± 4.29 cells/section, Fig. 4A, B). Similarly, nestin (21.24 ± 2.31 cells/section, Fig. 4C, D) and Ki‐67 (30.62 ± 18.78 cells/section Fig. 4E, F) immunoreactive cells were abundant in the SVZ of the control animals while a faint trace of nestin were detected mainly in ventricular lining of the lateral wall of the lateral ventricle and very few Ki‐67 immunoreactive cells could be observed in the SVZ region in 7‐6 h‐7 Ara‐C treatment group (0.19 ± 0.12 cells/section). Overall, immunofluorescent analysis demonstrated that intermittent Ara‐C treatment as applied in 7‐6 h‐7 Ara‐C kill paradigm could effectively deplete most of the neural stem and progenitor cells from the SVZ of the adult mouse brain.

## Discussion

We aimed to investigate the effects of intermittent Ara‐C infusion into the lateral ventricles of the adult mouse brain eliminating the entire NSCs population from the subventricular zone. Knowing that NSCs are usually in a dormant or quiescent status and divide only to maintain themselves and to repopulate the lost progeny in their niches, we took advantage of this notion to eliminate NSCs by recruiting them after depletion of their immediate progeny using the antimitotic drug Ara‐C. Our results demonstrated that by introducing a gap of 6 or 12 h in a 14 day Ara‐C kill paradigm, the number of NSCs would significantly decrease comparing to the 7 and 14 days continuous Ara‐C kill paradigms. Importantly despite severe reduction in stem and progenitor cells in all kill paradigms, none of the kill paradigms resulted in permanent depletion of NSCs and the SVZ niches repopulate via the escaped NSCs or from a more undifferentiated precursor cells in their developmental hierarchy.

Ara‐C infusion for 7, 14, and 14 days with 6 or 12 h of infusion gaps eliminated around 95%, 97%, 98%, and 99% of actively dividing cells from the subventricular zone of the lateral ventricle, respectively. Although these numbers seems to be very close, the small differences in percentages reflects the success of different kill paradigms in depletion of NSCs. Accordingly, the 7‐6 h‐7 and 7‐12 h‐7 kill paradigms turned out to be much more effective in elimination of NSCs than the 7 and 14 day continuous Ara‐C infusion. This is further evidenced by the statistically significant differences observed in neurosphere formation recovery rate 7 days post Ara‐C treatment in different kill paradigms (Fig. 1D). While the 7 and 14 days continuous Ara‐C infusion paradigms reached to almost 30% neurosphere‐forming frequency of the control group 7 days post Ara‐C infusion, the 7‐6 h‐7 and 7‐12 h‐7 paradigms could only reach to less than 15% of this value. This delay in neurosphere formation recovery in 7‐6 h‐7 and 7‐12 h‐7 kill paradigms is simply due to loss of a fair number of NSCs upon recruiting into the pool of actively dividing cells and subsequent depletion via re‐exposure to Ara‐C. Therefore, although the length of Ara‐C infusion is important to efficiently kill the stem and progenitor cells, for example, as evident in 14 days comparing to 7 days continuous Ara‐C infusion paradigm, the infusion gaps play a more important role as comparing the 7‐6 h‐7 and 7‐12 h‐7 to 14 days paradigms. Our results are in agreement with the study by Morshead at al., (Morshead et al. 1994) in which they showed that double kill paradigm using ^3^H‐Thymidine on day 0 and day 2 resulted in a 50% reduction in NSCs population that did not return to the control values even after 31 days post kill. Despite differences in the antimitotic agents (Ara‐C vs. ^3^H‐Thymidine) and time‐line of re‐exposure (2 days vs. 6 or 12 h after the initial exposure) in these two studies, we achieved similar results. This implies that at a given time point after the initial exposure not more than 50% of the dormant NSCs population become activated and hence not all NSCs could be eliminated using this double kill paradigms.

Long‐term proliferation or the ability to generate a large number of progeny is one of the cardinal features of NSCs upon which they could be distinguished from the other progenitor cells in a given neural cell population. Hence, neurospheres generated from stem cells are capable of generating new spheres upon replating and this can continue indefinitely (Reynolds and Weiss 1992; Deleyrolle et al. 2011). Interestingly, the neurospheres resulted from all kill paradigms in animals killed immediately after completion of Ara‐C infusion could not be serially passaged but neurospheres resulted from the animals killed 7 days after completion of Ara‐C infusion could be passaged several times and generate a large number of progeny in all Ara‐C kill paradigms (Fig. 2K). The inability to serially passage neurospheres resulted from the SVZs harvested immediately after Ara‐C infusion could be justified in two possible scenarios. First, long‐term exposure to Ara‐C could temporarily inactivate the cell cycle machinery of the remaining NSCs, hence, culturing the SVZ cells immediately after Ara‐C exposure in a proliferative condition may not result in generation of actively proliferative progeny to ensure serial passaging. This is evidenced by formation of very few small, compact and irregular‐shaped neurospheres in all Ara‐C‐treated groups (Fig. 2C‐F). These cells might need interactions with other cells in their in situ niches to receive appropriate signals in order to get reactivated and become fully functional. Support for this statement comes from the capability of the neurospheres from the cells harvested 7 days after completion of Ara‐C infusion from the SVZ of all kill paradigms. Second, long‐term exposure to Ara‐C could wipe out the majority of NSCs. In a study by Louis et al., (Louis et al. 2008), while developing the N‐CFCA, they found that 1 day infusion of 2% Ara‐C into the lateral ventricle resulted in a sharp decrease in progenitor‐derived colonies but not in stem cell–derived colonies. Increasing the Ara‐C infusion to three consecutive days resulted in a significant decrease in stem cell–derived colonies. Considering this study, we infused Ara‐C twice as long in the shortest kill paradigm, it is logical to expect a substantial loss of NSCs at least in 7‐6 h‐7 and 7‐12 h‐7 paradigms. The justification for acquiring long‐term passage capability of the resulting neurospheres 7 day after completion of Ara‐C could be due to acquiring full proliferative potential recovery of the remaining NSCs or emergence of new NSCs from a more undifferentiated precursor cells residing in the SVZ. In support of the latter hypothesis, it has recently been shown that after complete ablation of GFAP expressing type B adult NSCs in GFAP‐thymidine kinase transgenic mice (GFAP‐TK) using Ganciclovir (GCV), type B adult NSCs could be replenished from a very rare population of leukemia inhibitory factor (LIF) responsive primitive neural stem cells (pNSCs) (Sachewsky et al. 2014).

In line with our NSA results, immunofluorescent analysis on the SVZ of 7‐6 h‐7‐paradigm immediately after completion of Ara‐C infusion showed that all doublecortin‐positive type A cells and almost all of the actively proliferating Ki‐67‐positive cells and also nestin immunoreactive cells were depleted, whereas some GFAP‐positive cells were still present in the subventricular areas of the lateral ventricles. Of these GFAP‐positive cells a very small fraction could be dormant stem cells but it is very difficult and inaccurate to quantify the number of remaining NSCs based of GFAP immunoreactivity due to presence of other GFAP expressing astrocytes in subventricular zone. To circumvent this limitation, we performed N‐CFCA to ensure the presence or absence of these cells based on their functional criteria in generating large colonies as mentioned earlier. N‐CFCA of the cells harvested from the SVZ of the 7‐6 h‐7‐kill paradigm immediately after completion of Ara‐C infusion resulted in formation of very few small colonies that apparently were not derived from NSCs. On the basis of serial passaging capability of the neurospheres resulted from cells harvested from the SVZ of 7‐6 h‐7‐paradigm 7 days after completion of Ara‐C infusion, we expected to see large NSC‐derived colonies upon plating cells from this group. Surprisingly, although the number and size of colonies increased, we could find some relatively large colonies (between 1‐1.5 mm in diameter) that could serially propagate in vitro indicating the presence of NSCs in these colonies. This phenomenon could either be because of defective proliferation of the remaining NSCs due to long‐term Ara‐C exposure or is an indication for presence of new NSCs from the source of pNSCs. The latter might acquire more of adult NSC characteristics upon culturing and passaging in vitro to generate large number of progeny.

## Conclusions

This study clearly showed that implementing Ara‐C infusion gaps is very necessary to effectively eliminate NSCs through their recruitment into the actively dividing cell pool. Using this strategy, we could eliminate a fair number of the NSCs but it should be emphasized that this effect is temporary and the stem cell niches in subventricular zone of the adult mouse brain repopulate via the remaining NSCs or through a more undifferentiated precursor cells in their developmental hierarchy.

## Conflict of Interest

Authors declare that they have no conflict of interests.
